# Heart rate variability–guided endurance training: evaluating strengths, weaknesses, opportunities, and threats for load prescription and adjustment

**DOI:** 10.3389/fspor.2026.1858271

**Published:** 2026-08-27

**Authors:** Marcelle Schaffarczyk, Billy Sperlich

**Affiliations:** Integrative and Experimental Exercise Science and Training, Institute of Sport Science, Julius-Maximilians-Universität Würzburg, Würzburg, Germany

**Keywords:** adaptive training, decision-making, endurance, heart rate variability, HRV-guided training, load prescription

## Abstract

Heart rate variability (HRV) has been used to guide individualized endurance training prescription. By supporting training adjustments according to recovery status, HRV-guided approaches may improve the alignment between training load and physiological readiness. However, the practical value depends on the decision framework used to translate HRV measurements into training prescriptions. This paper applies a Strengths, Weaknesses, Opportunities, and Threats (SWOT) framework to critically evaluate the practical limitations of HRV-guided training regulation. Strengths include a well-established physiological rationale, evidence from controlled studies, expanding application across broader populations, and methodological advances in monitoring supported by wearable technologies. Weaknesses include the limited specificity of HRV as a global marker of cardiac autonomic modulation, the absence of standardized multimodal frameworks, and the persistent gap between research-based monitoring concepts and their implementation in practice. Opportunities lie in the development of transparent multivariate decision architectures, refinement of baseline and progression logic, and data-driven approaches to support individualized, context-aware training adjustments. Threats arise from rule-based decision frameworks that may inadequately capture the complexity and context-dependency of physiological adaptation, limited replication in real-world training environment, and growing dependence on proprietary metrics whose underlying algorithms lack transparency and may oversimplify training decisions. Overall, the primary limitation of HRV-guided endurance training is no longer the availability of physiological data, but the lack of transparent and physiologically grounded decision frameworks that define how HRV-derived signals should be interpreted, weighted, and translated into valid and actionable training prescriptions. Addressing this gap represents a critical step toward the development of adaptive training frameworks.

## 1 Introduction

Heart rate variability (HRV), reflecting the beat-to-beat fluctuations in the time interval between successive heartbeats, is widely used as a non-invasive marker of cardiac autonomic regulation and has been increasingly applied in athlete and patient monitoring and training prescription as an indicator of physiological stress and recovery status (1, 2). Beyond a simple representation of sympathovagal balance, HRV is increasingly understood as the result of complex and non-linear interactions among multiple regulatory mechanisms, including respiratory modulation, baroreflex function, central autonomic control, and intrinsic cardiac regulation (3–5). Within this framework, specific HRV-derived indices are used to operationalize these regulatory dynamics in applied settings. In particular, vagal-related indices such as the root mean square of successive differences (RMSSD) are considered sensitive to short-term changes in autonomic regulation and have been widely adopted in applied sport settings (2, 6–9) and clinical circumstances (1, 10–12).

**Figure 1:**
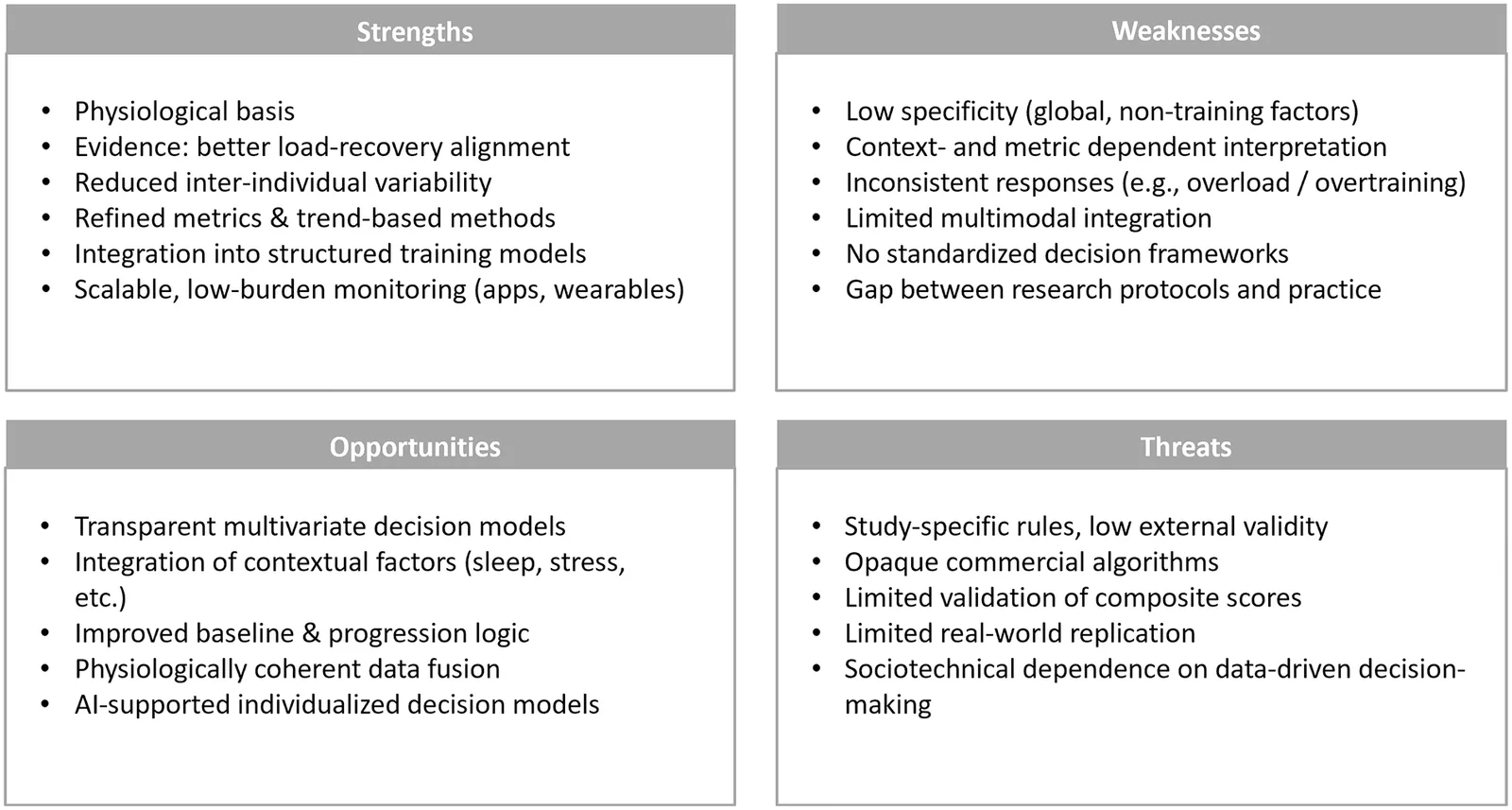
Strengths, weaknesses, opportunities, and threats associated with Heart Rate Variability–Guided Endurance Training.

Advances in wearable sensor technology and smartphone-based photoplethysmography (PPG) have facilitated the large-scale implementation of HRV monitoring in both research and applied contexts (13–15). As a result, HRV is increasingly incorporated into monitoring systems to support individualized training management, with controlled studies suggesting that HRV-guided load adjustments may improve the alignment between training demands and recovery status while reducing inter-individual variability in training adaptations (16, 17). The present analysis focuses specifically on HRV-guided endurance training, where the evidence base for HRV-guided load adjustment is currently most established.

Despite this growing application, the physiological interpretation and practical implementation of HRV in endurance training decision-making remain complex. HRV represents a global marker of autonomic regulation and is influenced by a wide range of physiological, behavioral, and environmental factors beyond exercise-related stress (2, 5, 18, 19). Furthermore, methodological differences in measurement protocols, data processing, and decision rules complicate comparability across studies and limit the generalizability of findings (11, 16).

At the same time, the increasing integration of HRV into commercial wearable technologies has led to the widespread use of composite readiness and recovery scores, which often function as black boxes due to proprietary algorithms with limited transparency and independent validation (20, 21). This development raises important questions regarding the interpretability, validity, and practical utility of HRV-derived decision-support in real-world endurance training environments.

In this context, a structured evaluation is required to critically assess both the scientific evidence and practical limitations of HRV-guided endurance training, thereby informing its strategic application. The present paper therefore applies a Strengths, Weaknesses, Opportunities, and Threats (SWOT) framework to systematically evaluate HRV as a basis for decision-making in individualized endurance training load prescription and adjustment, and to identify key directions for future methodological and applied development.

## 2 Strengths

### 2.1 Consolidated evidence from controlled trials

Two systematic reviews with meta-analyses published in 2021, each synthesizing eight controlled interventions, reported largely convergent findings regarding the effects of HRV-guided endurance training compared with predefined training programs (16, 17). Both reviews reported no significant advantage of HRV-guided prescription for maximum oxygen uptake (VO₂max) or endurance performance, with pooled effects remaining trivial to small across analyses (g = 0.079–0.171; SMD = 0.13–0.20). More favorable findings were observed for submaximal physiological adaptations. Düking et al. (17) reported a significant small-to-moderate benefit of HRV-guided training for threshold-related outcomes, including ventilatory and lactate threshold parameters (g = 0.296, 95% CI: 0.031–0.562; *p* = 0.028), although funnel plot asymmetry suggested potential publication bias. Similarly, Manresa-Rocamora et al. (16) reported consistently small-to-moderate effects favoring HRV-guided training across aerobic fitness outcomes, including peak power/velocity and ventilatory-threshold-related parameters (SMD = 0.20–0.44), although none of these pooled effects reached statistical significance. In addition, Manresa-Rocamora et al. (16) found significantly greater improvements in vagal-mediated HRV indices [RMSSD/Standard deviation 1 from the Poincaré Plot (SD1); SMD = 0.50, 95% CI: 0.09–0.91], indicating enhanced cardiac-autonomic adaptation under HRV-guided prescription. Beyond group-average effects, both reviews noted evidence from individual studies suggesting fewer negative responders and a more homogeneous distribution of physiological and performance adaptations in HRV-guided groups. Although the available evidence is limited by relatively small samples and predominantly short interventions of up to eight weeks, the convergence of findings across independent meta-analyses suggests that HRV-guided load regulation in endurance training may primarily improve the consistency of training adaptation and cardiac autonomic regulation, with the most consistent benefits observed in submaximal physiological and vagal-autonomic markers rather than maximal aerobic capacity or endurance performance.

### 2.2 Methodological advancements

Early HRV-guided endurance training models adjusted training intensity according to deviations from an individual HRV baseline (22, 23). In these frameworks, daily HRV values were compared against a rolling reference derived from preceding measurements (typically spanning up to 10 days), with decision rules incorporating both central tendency and variability to guide training adjustments. When HRV fell below this individualized threshold—indicating elevated physiological strain—the initially pre-planned number of high-intensity sessions was reduced, postponed, or replaced by low-intensity exercise or recovery, whereas stable or increased HRV permitted higher-intensity training. Because these early approaches relied on single-day measurements, subsequent methodological developments shifted toward trend-based interpretations to enhance signal stability and reduce susceptibility to transient physiological variability. Current recommendations therefore emphasize rolling baseline approaches, typically using 7-day moving averages derived from repeated measurements per week and interpreted relative to individualized “normal ranges” such as the smallest worthwhile change (SWC), which is commonly derived from the mean and variability of consecutive baseline recordings (2, 5, 18, 24–26). Into this matter, methodological work increasingly emphasizes the importance of standardized measurement procedures, including consistent body position, timing, and recording conditions, because these factors substantially affect HRV values and their longitudinal interpretability (5, 16, 24). For example, morning assessments under standardized conditions are commonly recommended to minimize the influence of transient behavioral and environmental stressors on repeated measurements (24, 27). For comprehensive methodological guidance on HRV monitoring, including data acquisition, recording procedures, signal processing, interpretation, and practical implementation, readers are referred to the methodological reviews cited herein (2–5, 24, 27).

In parallel, methodological HRV metric refinement has occurred as well. Whereas early studies employed heterogeneous indices such as high-frequency (HF) power or SD1 (22, 23), contemporary monitoring frameworks increasingly favor lnRMSSD due to its greater robustness and superior short-term reliability in field-based monitoring (2, 5, 16). Complementary physiological indicators such as resting heart rate (HR) may provide additional context for interpreting HRV fluctuations. While lnRMSSD primarily reflects vagally mediated beat-to-beat variability, resting HR (or the prevailing RR interval) may help distinguish sympathetic activation from vagal saturation effects. Accordingly, joint interpretation of lnRMSSD with HR- or RR-derived metrics (e.g., lnRMSSD/RR) has been proposed to reduce ambiguity in autonomic interpretation (2, 5, 6, 18, 24, 25). Another complementary marker is the coefficient of variation (CV) of lnRMSSD (lnRMSSD_CV_), which reflects day-to-day fluctuations in vagally mediated HRV and provides additional insight into short-term autonomic perturbations and recovery status beyond lnRMSSD. Favorable training adaptation is generally characterized by an increase in lnRMSSD together with low or reduced lnRMSSD_CV_ (8, 27, 28). However, lnRMSSD_CV_ should always be interpreted alongside lnRMSSD and the training context, as transient elevations may reflect acute homeostatic perturbations during intensified training, whereas persistent alterations may indicate maladaptive responses depending on the stage of adaptation (2, 18, 27, 29). Although current HRV-guided endurance training interventions are almost exclusively based on resting HRV measurements, assessment of cardiac autonomic responses to standardized physiological challenges has emerged as a complementary approach to characterize autonomic reactivity beyond resting conditions (1). Such challenges include orthostatic testing (22, 24) as well as standardized submaximal exercise (30, 31), the latter enabling the assessment of exercise-derived HRV. Within this context, detrended fluctuation analysis alpha-1 (DFAa1) has recently emerged as a promising marker of systemic regulation, extending HRV assessment beyond resting measurements and providing additional insight into fatigue-related alterations in cardiac autonomic control (5, 30, 32). However, despite increasing interest in lnRMSSD_CV_ and DFAa1 as markers of recovery stability and exercise-related cardiac autonomic regulation, respectively, their application within HRV-guided training interventions remains largely unexplored. To date, HRV-guided training studies have almost exclusively based training decisions on resting vagal-related indices (RMSSD, SD1, HF) leaving the potential utility of alternative HRV markers for individualized load regulation largely unknown.

Finally, while most HRV-guided interventions primarily modified training intensity in response to changes in autonomic status (22, 23, 33–37), a more recent approach has expanded this principle to broader training-load management. Nuuttila et al. (38) embedded HRV-guided decision-making within a periodized training program and adjusted overall training load according to recovery status across preparatory, volume-focused, and interval-focused phases. In contrast to earlier intensity-based models, training prescriptions involved increases, maintenance, or reductions in overall training load, including modifications of training volume during the volume-focused phase and adjustments in the frequency of high-intensity interval sessions during the interval-focused phase. This represents one of the first attempts to extend HRV-guided endurance training prescription beyond intensity regulation toward broader training-load management. However, the training adjustments remained phase-specific, preventing the independent evaluation of volume- and intensity-related load manipulations. Consequently, it remains unclear which specific HRV-guided endurance training adjustments were primarily responsible for the observed benefits, as modifications to training volume and the frequency of high-intensity sessions were implemented during different phases of the program. More broadly, evidence remains limited regarding how different dimensions of training load should be weighted and integrated within individualized decision-making frameworks. Furthermore, existing HRV-guided interventions have generally retained predefined training frameworks, with only limited flexibility in the scheduling and organization of training sessions (22, 23, 33–39). As a result, it remains unclear not only which dimensions of training load should be individualized, but also how much flexibility should be incorporated into HRV-guided endurance training systems to accommodate individual preferences, scheduling constraints, and day-to-day decision-making.

### 2.3 Expansion beyond athletic populations

To date, evidence for HRV-guided endurance training has been derived predominantly from endurance-trained or recreationally active populations, with female participants often being underrepresented (17). By contrast, evidence in non-athletic and clinical populations has remained comparatively limited. Although an early proof-of-concept study applied HRV-guided exercise prescription in patients with ischemic heart failure, using daily adjustments of exercise intensity and duration based on multiple time-domain HRV indices, the intervention relied on a short three-day baseline and day-to-day HRV comparisons, reflecting an early stage in the methodological development of HRV-guided training (12).

More recently, randomized trials have extended HRV-guided training to broader populations. For example, HRV-guided training has been successfully implemented in previously sedentary men and women using both trainer-guided and app-based approaches. In an 11-week program combining strength and aerobic interval training performed three times per week, both delivery modes improved VO₂peak, exercise-test duration, muscular strength, and vagal-related HRV indices, while adherence remained comparable between groups (39). Furthermore, in cardiac rehabilitation, HRV-guided interval training achieved similar improvements in VO₂max and metabolic equivalents as traditional high-intensity interval training while requiring less high-intensity exercise volume and eliciting more favorable cardiovascular responses (40). Similarly, a randomized trial in patients with coronary artery disease reported superior improvements in vagal-related HRV following HRV-guided training compared with predefined exercise prescription, although improvements in VO₂max were comparable between groups (41).

Despite these promising findings, evidence in clinical populations remains sparse. Available studies suggest that population characteristics and clinical factors may substantially influence HRV responses and training adaptations. Factors such as age, sex, physical fitness, disease characteristics, treatment modalities, and medication use have all been proposed as potential determinants of cardiac autonomic responses in clinical settings (11). Consequently, further research is required to determine whether HRV-guided endurance training principles generalize across clinical and health-oriented exercise contexts.

### 2.4 Field feasibility and accessibility

Validated smartphone applications [e.g., HRV4Training (14), Welltory (42), Elite HRV (43)] and wearable devices, including chest straps [e.g., Polar H10 sensor (44) or Movesense Medical (45)], wrist-worn devices [e.g., Apple Watch Series 6 (46, 47)], or ring-based sensors [e.g., Oura (47, 48)] have made daily HRV monitoring feasible outside well-controlled laboratory settings. Although PPG-based wearables, such as wrist-worn or ring-based devices, have substantially improved the accessibility of longitudinal monitoring, PPG-derived pulse rate variability should not be considered fully interchangeable with electrocardiogram (ECG)-derived HRV, the gold-standard method for assessing beat-to-beat cardiac intervals, because pulse rate variability reflects the arrival of pulse waves at the periphery rather than the timing of cardiac electrical activity and may therefore be affected by pulse transit time, peripheral vascular processes, movement artifacts, and recording conditions (49, 50). Accordingly, ECG remains the preferred method when high-fidelity beat-to-beat interval assessment or clinical evaluation (e.g., ectopic beat detection or arrhythmia screening) is required, whereas PPG-based devices may provide acceptable estimates for standardized longitudinal monitoring under resting conditions (47, 51). While error may vary according to the HRV metric, body position, and biological sex (51), as well as device-specific measurement windows and proprietary signal-processing algorithms (47), portable devices remain practical tools for longitudinal field-based monitoring. Compared with traditional laboratory approaches, these technologies reduce measurement burden and facilitate routine data collection in applied settings, thereby supporting the practical implementation of HRV-guided endurance training (36, 40). Moreover, contemporary wearables increasingly capture context-relevant signals (e.g., accelerometry, temperature), yet these data are rarely integrated into HRV-based decision frameworks (52).

## 3 Weaknesses

### 3.1 Limited dimensionality of HRV as a single marker

HRV is an indirect marker of cardiac autonomic modulation rather than a direct measure of autonomic nerve activity, and its physiological interpretation depends strongly on the measurement context and the specific HRV metric used (3, 5). Accordingly, HRV should be regarded as a global and non-specific marker of cardiac autonomic regulation rather than an isolated indicator of exercise-related stress (2, 4). This is further complicated by the fact that HRV reflects the combined influence of multiple interacting regulatory mechanisms, such as respiratory sinus arrhythmia and baroreflex function, which are typically not disentangled in applied monitoring contexts (24, 52). Large-scale analyses demonstrate that HRV responds to numerous non-exercise stressors, including illness, alcohol intake, and menstrual cycle phase (19). Additionally, more recent dense longitudinal monitoring data demonstrated significant associations between day-to-day HRV fluctuations and subjective wellbeing in naturally cycling women (53). Evidence from clinical intervention studies further supports this limited specificity, showing that HRV is modulated by a wide range of non-exercise stimuli, including pain-modulating therapies, independent of training-related stress (54). Measurement conditions (e.g., measurement methodology, sampling rate, or electrophysiological signal quality), behavioral factors (e.g., sleep, hydration, prior exercise), and environmental stressors (e.g., noise, temperature, light) further contribute to variability in HRV recordings and complicate interpretation in applied monitoring contexts (2, 5, 18, 25, 55). In endurance athletes, short-term changes in physiological state—such as fluctuations in blood plasma volume—may also substantially affect HRV measurements (18). Consequently, changes in HRV do not necessarily indicate meaningful alterations in fatigue, fitness, or performance capacity, as similar HRV patterns have been associated with both positive and negative training outcomes (6, 18, 28). Accordingly, higher HRV should not be interpreted as inherently reflecting a more favorable physiological state, as increases in HRV have been associated with both improved performance and functional overreaching depending on the physiological context (6, 18, 28). Beyond this limited specificity, HRV primarily reflects cardiac autonomic regulation and cardiovascular recovery but is less informative for other aspects of post-exercise recovery, such as restoration of muscle glycogen stores or repair of damaged muscle tissue (56), and therefore cannot capture the multidimensional nature of recovery processes occurring across physiological systems. Consistent with this, divergences between HRV and perceptual or neuromuscular recovery have been reported (57–59), further indicating that HRV alone provides an incomplete basis for decisions on training load and recovery prescription. This limitation aligns with broader consensus statements in athlete monitoring, emphasizing that no single marker can adequately capture the complex and multidimensional interactions underlying fitness, fatigue, and recovery processes (60, 61).

### 3.2 Limited evidence for structured multimodal integration

Although athlete status is increasingly recognized as a multidimensional construct (60), explicit multimodal decision schemes remain uncommon. Early approaches, such as the protocol by Capostagno et al. (62), combined physiological indicators derived from the Lamberts and Lambert Submaximal Cycle Test, including HR and HR recovery, with perceptual ratings of exertion within predefined decision rules to regulate training. However, such implementations remain highly study-specific, with fixed criteria and heuristic decision rules—often selected for practical feasibility, rapid field-based application, and known day-to-day variability—rather than transparent frameworks specifying how individual markers should be weighted, prioritized, or integrated.

Within the context of HRV-guided training, Nuuttila et al. (38) combined nocturnal HRV with perceived recovery and a HR–running speed index to adjust training load across structured training phases. While this approach demonstrates the potential of multimodal monitoring, the authors explicitly highlighted the lack of research on how such multi-parameter approaches can be operationalized in practice. This limitation is consistent with broader athlete-monitoring research indicating that subjective and objective measures often provide distinct and only partially overlapping information about athlete status and may therefore serve complementary monitoring roles (61). However, the practical implementation of multidimensional monitoring remains challenging, as practitioners must balance scientific complexity with operational feasibility while establishing a clear rationale for selecting monitoring variables, interpreting the resulting data, and translating observations into actionable decisions (63, 64). Consequently, existing multimodal approaches remain heterogeneous and largely protocol-specific, with limited standardization and external validation. To date, no broadly applicable framework that systematically integrates cardiac autonomic, perceptual, and performance-based indicators into a coherent and transparent decision-making architecture has been established.

### 3.3 Research-application disconnect

Although controlled trials have implemented structured HRV-guided decision protocols using validated mobile applications or wearable devices, the interpretation of HRV data—such as rolling averages, SWC thresholds, or subsequent training-intensity adjustments—typically relies on predefined research protocols rather than automated app-driven decision logic (34, 36, 40). For example, in randomizeded trials (36, 40) using the HRV4Training platform, daily lnRMSSD values were obtained via smartphone-based measurements, yet training adjustments were implemented according to externally defined decision rules specified in the study design rather than through automated in-app prescription. Similarly, da Silva et al. (34) implemented HRV-guided training using RMSSD derived from RR intervals recorded with a HR monitor and analyzed in Kubios HRV software (65), but the resulting values still required manual interpretation and application of predefined decision rules by the investigators. Accordingly, digital tools in these studies primarily functioned as data acquisition and analysis interfaces, whereas the actual load-regulation logic remained researcher-defined and manually implemented.

A small number of studies have incorporated a greater degree of automation. For example, Nuutilla et al. (35) used a proprietary recovery score derived from HR and RMSSD to determine progression between predefined high-intensity training blocks, while Casanova-Lizon et al. (39) integrated a predefined HRV-guided algorithm directly into a mobile application that automatically translated HRV measurements into individualized training recommendations and progression levels within a combined strength and aerobic interval training program. Nevertheless, these approaches differ substantially in their transparency. In Nuutilla et al. (35), training decisions relied on a proprietary recovery score that was adaptively scaled according to each participant's measurement history. As the underlying calculation procedure was not fully described, it remains difficult to determine how physiological information was weighted and translated into training recommendations. By contrast, the decision rules implemented by Casanova-Lizon et al. (39) were predefined and described more explicitly, but were evaluated within a specific pilot application and training context. Consequently, the broader generalizability and external validation of such automated HRV-guided decision systems remain limited.

This challenge extends beyond experimental settings. As highlighted by Düking et al. (20), many contemporary wearable systems primarily operate at the level of data collection, monitoring, and insight generation, while providing limited transparency regarding the evidence base and decision logic underlying training recommendations. In parallel, commercially available platforms frequently generate composite readiness or recovery indices (21) derived from longitudinal HRV patterns. However, these outputs generally provide generalized guidance rather than transparent, evidence-based frameworks specifying how exercise intensity or training volume should be systematically modified. While some systems have begun incorporating resting HRV and DFAa1-based prediction models (66), their decision logic and weighting strategies are typically proprietary and not publicly disclosed. Consequently, the extent to which autonomic indicators are systematically integrated within decision architectures remains unclear, creating a structural gap between experimentally validated HRV-guided training protocols and their standardized implementation in applied practice.

## 4 Opportunities

### 4.1 Development of structured and transparent decision architectures

Recent developments in wearable sensing, digital monitoring platforms, and AI-supported data processing create an opportunity to move beyond single-marker HRV interpretation toward structured, multi-signal decision frameworks (52, 67). Rather than merely aggregating data into summary scores, such frameworks should explicitly define the transition from data collection to information, insight, judgement, and training decision-making (20). This distinction is important because current consumer-oriented composite scores already combine multiple physiological and behavioral inputs, but often differ substantially in data collection windows, metric weighting, and scoring methodology, with limited disclosure of algorithms and insufficient independent validation (21). Future HRV-guided systems could therefore build on multi-signal integration while avoiding reliance on opaque “readiness” indices by specifying which signals are included, how they are weighted, under which conditions they are considered valid, and how conflicting indicators are resolved.

Within such a framework, HRV could be integrated with complementary signals such as accelerometry, sleep, perceptual recovery, training load, and, where feasible, respiratory or blood-pressure-derived information to improve the physiological specificity of fatigue and recovery interpretation (52). Rather than treating “readiness” indicators as a single output, such architectures could distinguish between training load, athlete state, and training response, using individualized baselines, distribution-based thresholds, and context-aware interpretation to support decisions that are both scientifically grounded and practically feasible (64). This creates a clear opportunity to develop transparent, physiologically grounded, and externally validated decision frameworks that formalize how autonomic, perceptual, behavioral, and performance-related signals should interact within adaptive training prescription, while retaining human oversight where uncertainty, contextual constraints, or athlete preferences require interpretation.

### 4.2 Refinement of baseline and progression logic

Adaptive HRV-guided training models typically regulate training load according to observed HRV (trends) in comparison to individualized HRV reference values (16, 17). However, considerable methodological heterogeneity exists regarding whether training decisions are based on single-day HRV measurements or averaged HRV values and how these values are interpreted relative to fixed or rolling reference criteria (16). Studies using rolling-averaged HRV values typically compared these values against fixed reference criteria derived from a multi-week baseline period that were either maintained throughout the intervention (35, 39), recalculated at predefined intervals (33, 36, 37), or continuously updated using rolling reference windows (38). In contrast, studies using single-day HRV measurements generally relied on continuously updated rolling reference criteria derived from preceding observations (22, 23, 34), although alternative approaches have also been proposed, including day-to-day comparisons against the immediately preceding measurement (68). While rolling-averaged HRV values may reduce sensitivity to transient day-to-day fluctuations and result in fewer training modifications, rolling reference criteria may better reflect ongoing physiological adaptation and current training responses (16). However, such reference limits may also gradually shift over time. As highlighted by Nuuttila et al. (38), such shifts can result in unintended upward or downward adjustments of decision thresholds, potentially affecting the stability and interpretability of training decisions. A key unresolved issue is therefore how reference values should be updated to balance responsiveness to short-term physiological changes with the maintenance of consistent decision criteria over longer training periods.

Despite increasing methodological refinement, no consensus currently exists regarding the optimal strategy for establishing, updating, and interpreting individualized reference values in HRV-guided training. Future research should therefore focus on systematically evaluating different combinations of averaging and reference strategies, including alternative approaches for updating individualized baselines over time. Such approaches may help balance sensitivity to short-term physiological fluctuations with the need for stable decision criteria over longer training periods, while reducing the risk of unintended shifts in decision thresholds as training adaptations occur (38). In addition, future research should establish transparent and reproducible procedures for defining, updating, and interpreting individualized reference values. Such standardization may improve the consistency, reproducibility, and interpretability of HRV-guided training prescriptions.

### 4.3 Context-aware and physiologically coherent signal integration

Because HRV reflects global cardiac autonomic modulation rather than training-specific recovery alone (2, 4, 19), its interpretation requires appropriate contextualization. This is consistent with consensus statements emphasizing that data interpretation rather than data collection represents primary challenge in athlete monitoring (60). In this context, the integration and interpretation of heterogeneous data streams has been identified as a major challenge, requiring interdisciplinary expertise to ensure meaningful and physiologically coherent conclusions (52, 67). Integrating structured contextual modifiers such as sleep quality, illness symptoms, menstrual cycle phase, or perceived stress, may improve interpretive specificity and reduce ambiguity in prescriptive decisions. However, recent evaluations of consumer composite health scores indicate potential redundancy and multicollinearity when closely related variables (e.g., sleep and HRV) are combined without physiologically informed weighting (21). An important opportunity therefore lies in developing integration frameworks that explicitly distinguish behavioral drivers from physiological responses, avoid double-counting correlated signals, and define how contextual information modifies the meaning of autonomic markers. Recent advances in artificial intelligence and machine learning may support this process by enabling the integration and automated analysis of multimodal data streams generated by wearable technologies (20, 52). Given the complex and nonlinear interactions among physiological subsystems underlying training adaptation (69), machine learning approaches may help identify patterns and relationships within multidimensional datasets that may be difficult to capture using traditional rule-based or linear modelling approaches (70). Such approaches may support the integration of physiological and contextual data streams and thereby improve the interpretation of monitoring signals within complex training environments.

## 5 Threats

### 5.1 Rule-based decision frameworks and limited external validation

Adaptive training protocols often rely on predefined decision rules based on deviations from individual HRV baselines to adjust training. While such rule-based approaches can demonstrate effectiveness within controlled interventions, their broader applicability remains uncertain. Systematic reviews of HRV-guided training studies highlight considerable methodological variability in the decision frameworks used to regulate training load, including differences in HRV indices, baseline reference calculations, and criteria used to trigger training adjustments across studies (16). Importantly, the definition of meaningful HRV deviations used to guide training decisions remains methodologically challenging. Existing HRV-guided interventions have employed a variety of approaches to define individualized decision boundaries, including reference ranges based on the individual mean ± 0.5 SD (36, 37, 39), rolling reference ranges based on a 4-week average ± 0.5 SD (38), standard deviation-derived thresholds relative to baseline values (22, 23, 34), relative percentage-change criteria based on day-to-day HRV fluctuations (68), and proprietary recovery-score algorithms (35). Consequently, similar HRV responses may result in different training recommendations depending on the analytical framework used to interpret the data (55). However, these approaches share a common characteristic: all rely on predefined decision rules that translate physiological measurements into specific training recommendations. No consensus currently exists regarding which of these decision frameworks most accurately reflects meaningful physiological change or provides the most appropriate basis for training prescription. More fundamentally, predefined rule-based systems may struggle to accommodate the complexity, variability, and context-dependency of physiological adaptation, particularly when applied across different athletes, training phases, and contexts (55, 69). This limitation is consistent with broader critiques in athlete monitoring, where commonly applied load-response models have been shown to oversimplify complex performance dynamics and exhibit limited predictive accuracy in applied settings (60). As a result, training decisions based solely on predefined thresholds may oversimplify the complex and context-dependent nature of physiological responses and lead to inappropriate adjustments of training load. Emerging data-driven approaches may help account for these complex interactions and support more individualized decision frameworks in athlete monitoring (20, 70).

### 5.2 Algorithmic opacity and commercial composite scores

Consumer-oriented readiness and recovery scores are increasingly used to guide training decisions, yet their underlying algorithms and weighting strategies remain largely undisclosed (21). In many commercially available systems, multiple physiological and behavioral inputs are aggregated into composite scores, while the specific contribution and interaction of individual variables remain opaque (21). While earlier work primarily highlighted limitations related to measurement validity and data quality in wearable technologies (13), more recent developments have shifted the focus toward the challenge of translating these data into evidence-based decision-making frameworks (20). Accordingly, a central limitation lies not only in data acquisition but also in how physiological and behavioral data are operationalized into actionable training recommendations. More broadly, translating wearable-derived data into physiologically meaningful and actionable insights remains a major unresolved challenge, further complicating the implementation of valid decision-support systems (67). Current AI-enabled wearable systems often provide automated outputs or generalized guidance without a clearly defined or scientifically grounded decision logic, limiting insight into how monitoring data are interpreted and applied (20, 52). A further concern relates to the limited independent validation of such composite decision models. Although wearable technologies may provide acceptable validity for individual physiological measurements, the transformation of these signals into composite readiness or recovery scores remains insufficiently validated, both in terms of reflecting underlying physiological states and in terms of their effectiveness as decision-support tools for training prescription (13, 15, 21). Consequently, it is often unclear whether these scores accurately represent athlete status or meaningfully support training-related decision-making. Taken together, the combination of limited transparency, structurally ambiguous score construction, unclear decision logic, and insufficient validation restricts interpretability and may lead to the widespread adoption of decision-support systems that are not fully aligned with current scientific evidence, potentially widening the gap between evidence-based training principles and applied practice.

### 5.3 Limited replication and context specific evidence

Multimodal and adaptive training protocols have demonstrated promising effects in controlled settings; however, the empirical evidence base remains limited. Systematic reviews of HRV-guided endurance training interventions highlight the relatively small number of studies, typically involving small sample sizes, short intervention durations, and specific populations (16, 17). These characteristics limit generalizability and replication across independent cohorts, performance levels, and sporting disciplines. In addition, many interventions have been conducted under tightly controlled experimental conditions that may not fully reflect real-world training environments. In applied contexts, training decisions are influenced by fluctuating schedules, travel, illness, psychosocial stressors, and varying adherence to monitoring protocols, all of which may alter physiological responses and data quality (61, 63). Furthermore, training adaptation is characterized by complex and non-linear interactions among physiological systems, which are highly individual and context-dependent (69). Consequently, adaptive systems that perform well in controlled trials may not retain the same level of effectiveness or stability in everyday training practice. Further research is therefore needed to examine these approaches in longer-term, ecologically valid settings and across more heterogeneous athlete populations.

### 5.4 Sociotechnical dependence

Because HRV-guided training requires repeated technology-mediated feedback, decision-making may become increasingly dependent on digital measurements, platform outputs, and continuous data availability. However, this development should not be understood as a property of HRV monitoring itself, but rather as part of a broader toward datafication of health, in which physiological and behavioural processes are increasingly translated into quantifiable data to guide decision-making across clinical care, self-care, and performance settings (71). Within sport, socio-technical analyses of elite performance environments have further highlighted how highly data-driven cultures may privilege indicators over coaching judgement, athlete self-report, or experiential knowledge, thereby reinforcing reliance on measurable information during training-related decision-making (72). Against this background, HRV-derived metrics should be considered as one component of a broader decision-making process rather than authoritative indicators of training readiness. Overreliance on HRV-derived outputs or algorithmic recommendations may inadvertently constrain training decisions to what can be quantified, even though training readiness and adaptation remain inherently context-dependent and only partially captured by physiological monitoring.

## 6 Summary

HRV-guided endurance training prescription represents a meaningful advancement in individualized load regulation, with controlled evidence indicating improved consistency of training adaptations compared with predefined programs. Methodological refinements and increasing field feasibility have further strengthened its practical relevance. However, the primary limitation of HRV-guided endurance training is no longer the availability of physiological data, but the interpretation, weighting and translation of these signals into valid and actionable training decisions. HRV reflects a global and context-sensitive marker of cardiac autonomic regulation, and current adaptive protocols rely largely on study-specific decision rules that lack standardization, transparency, and external validation. At the same time, commercially implemented readiness and recovery scores are increasingly shaping applied practice despite limited disclosure of their underlying algorithms and insufficient independent validation. This creates a structural gap between experimentally validated HRV-guided endurance training concepts and their real-world implementation in athlete monitoring systems. Consequently, the central challenge for the field is not whether HRV provides useful information, but how HRV-derived signals can be embedded within transparent, context-aware, and physiologically coherent decision frameworks. Future developments should therefore prioritize the integration of multimodal data, explicit decision architectures, and externally validated models that define baseline updating strategies and preserve progressive overload while accounting for individual and contextual variability. Addressing this research–application gap represents a critical step toward the development of robust and generalizable adaptive endurance training frameworks and defines the next phase in the evolution of individualized endurance training prescription.

## Data Availability

All information supporting the findings of this study is available in the published literature cited in the article.
